# Management of Right-Sided Cardiac Masses With Manual Aspiration Using AlphaVac Device: A Single-Center Case Series

**DOI:** 10.1016/j.jscai.2025.104160

**Published:** 2026-01-15

**Authors:** Yara Deeb, Rami Al-Ayyubi, Mohammed Martini, Abubaker Kallan, Rajanbir Singh, Vidya Sundareshan, Abdul Moiz Hafiz

**Affiliations:** a Department of Internal Medicine, Southern Illinois University School of Medicine, Springfield, Illinois; b Division of Cardiology, Southern Illinois University School of Medicine, Springfield, Illinois; c Division of Infectious Diseases, Southern Illinois University School of Medicine, Springfield, Illinois

**Keywords:** AlphaVac, case report, central line-associated thrombosis, infective endocarditis, lead-associated endocarditis, percutaneous mechanical aspiration, right-sided cardiac masses

## Abstract

Right-sided cardiac masses pose therapeutic challenges, particularly in patients at high surgical risk. The AlphaVac mechanical aspiration system (AngioDynamics) provides a minimally invasive, endovascular alternative to surgery or AngioVac (AngioDynamics). We report 6 consecutive patients managed with AlphaVac, all deemed poor surgical candidates by a multidisciplinary heart team. One patient died from severe comorbidities and persistent fungemia despite aspiration. Four achieved sufficient debulking, allowing stabilization or definitive antimicrobial therapy when infected. One required transfer for valvular replacement due to aortic valve endocarditis with perforation and systemic embolism. AlphaVac enabled effective aspiration in high-risk patients unsuitable for immediate surgical intervention.

## Introduction

Right-sided cardiac masses are rare. Infective endocarditis (IE) vegetations and thrombi account for most cases. Surgical intervention during the active phase of IE is associated with a mortality rate of 6% to 10%, with poor outcomes in older patients, those with mycotic infection, intravenous drug users, and patients with tricuspid valve replacement or intracardiac devices.^1^ Surgical timing remains controversial and is typically postponed until after infection has been controlled,^2^ often after 4 to 6 weeks of antibiotics.

Percutaneous mechanical aspiration (PMA) provides a less invasive approach for critically ill patients as an adjunctive infection control strategy. Our case series explores large-bore PMA strategies with AlphaVac (Angiodynamics) for right atrial thrombi and IE-related masses. A structured case series presentation is provided in Table 1.

**Table 1:** Clinical characteristics, procedural details, and outcomes of 6 consecutive patients who underwent AlphaVac aspiration between 2023 and 2025.

Case	Age, y	Sex	PMH	PSHx	HPI	Investigation	Indications	Procedure	Postprocedural course
Case 1	39	F	Ulcerative colitis, stage IV colorectal adenocarcinoma, recurrent GI bleed, chronic TPN	Total colectomy, end ileostomy	Persistent fungemia (*Candida glabrata*), PE, recurrent GI bleeding requiring multiple PRBC transfusions, AKI requiring CRRT, SBO	TEE: Mobile linear echogenic structure measuring 3.9 cm in SVC.	• Persistent fungemia • Active GI bleed • Reduce need for anticoagulation	RCFV: 2 vegetations removed from SVC, measuring 1 × 15 cm and 0.25 × 7 cm. Only 5000 units of heparin were used with an intentional subtherapeutic goal.	The family chose comfort measures; she passed away in the hospital.
Case 2	62	M	High-degree atrioventricular block, PE	Biventricular pacemaker	Bacteremia (*Streptococcus agalactiae*), hemorrhagic shock	TEE: A 4-cm septal tricuspid valve mass and a 2.8-cm right atrial mass arising from the Eustachian valve.	• Lead-associated endocarditis • Septic, hemorrhagic shock • PE	RIJ: the mass was removed.	Transferred for laser lead extraction.
Case 3	45	F	Chronic TPN, recurrent bacteremia	Roux-en-Y	Septic shock, MSSA bacteremia	TEE: A 1.7-cm mass in the SVC and a 4.3-cm mass along the inferior right atrial wall, prolapsing through the tricuspid valve into the right ventricle, causing severe TR.	• Persistent bacteremia • Mass location precluding central cannulation	RCFV: unsuccessful secondary to interrupted IVC with azygous continuation. RIJ: A 1.5-cm residual mass attached to the right atrial free wall was not removed due to concern of atrial free wall rupture.	TTE in 6 mo: improvement of TR to mild–moderate.
Case 4	68	F	Hypertension, hyperlipidemia, CAD	—	Purulent peritonitis, perforated diverticulitis required resection and colostomy	TTE: A 1.8 × 1.3 cm mass attached to the central line with mobile component.	• Central line-associated thrombosis • Bowel perforation	RCFV: 1.6-cm mass extracted; no residual thrombus was noted.	Clinical improvement.
Case 5	38	M	ESRD on HD, tunneled dialysis line vegetation, hypertension, type 2 diabetes mellitus, MRSA bacteremia, PFO	Unilateral below-the-knee amputation due to osteomyelitis	Persistent MRSA bacteremia, acromioclavicular joint septic arthritis, periarticular abscess, septic emboli	TEE: A 2.66 × 1.27 cm SVC mass extending to the right atrium, partially obstructing SVC outflow. Another 0.64 × 0.60 cm mass at the right atrium superior posterior wall.	• Persistent bacteremia • SVC obstruction • Septic emboli • ESRD • PFO	RCFV: thrombectomy of the right atrial mass was successful; SVC debulking was unsuccessful due to chronic fibrotic change. Final angiography confirmed SVC patency.	Surgical repair of chronic SVC narrowing deferred.
Case 6	50	M	IVDU	—	Severe sepsis, MSSA bacteremia, cellulitis, acute anemia, thrombocytopenia, septic emboli, splenic infarct	TTE: A 2.4-cm tricuspid posterior leaflet vegetation with moderate TR and possible mitral involvement. TEE: Deferred due to esophageal stenosis requiring balloon dilation.	• Persistent bacteremia • Septic emboli • IVDU • Acute anemia	RIJ: ICE/TEE during thrombectomy showed a 3.1 × 1.7 cm tricuspid vegetation; near complete debulking. Residual 1.5-cm thin serpiginous structure with moderate–severe TR, and a newly discovered 1.2 × 1.0 cm aortic vegetation concerning for cusp perforation.	Due to left-sided valve perforation and high embolic risk, he was referred to a quaternary center for surgical valve replacement.

AKI, acute kidney injury; CAD, coronary artery disease; CRRT, continuous renal replacement therapy; ESRD, end stage renal disease; F, female; GI, gastrointestinal; HD, hemodialysis; HPI, history of present illness; ICE, intracardiac echocardiography; IV, intravenous; IVC, inferior vena cava; IVDU, intravenous drug use; M, male; MRSA, methicillin-resistant *Staphylococcus aureus*; MSSA, methicillin-susceptible *Staphylococcus aureus*; PE, pulmonary embolism; PFO, patent foramen ovale; PMH, past medical history; PRBC, packed red blood cell; PSHx, past surgical history; RCFV, right common femoral vein; RIJ, right internal jugular; SBO, small bowel obstruction; SVC, superior vena cava; TEE, transesophageal echocardiogram; TPN, total parenteral nutrition; TR, tricuspid regurgitation; TTE, transthoracic echocardiogram.

## Discussion

**Figure 1:**
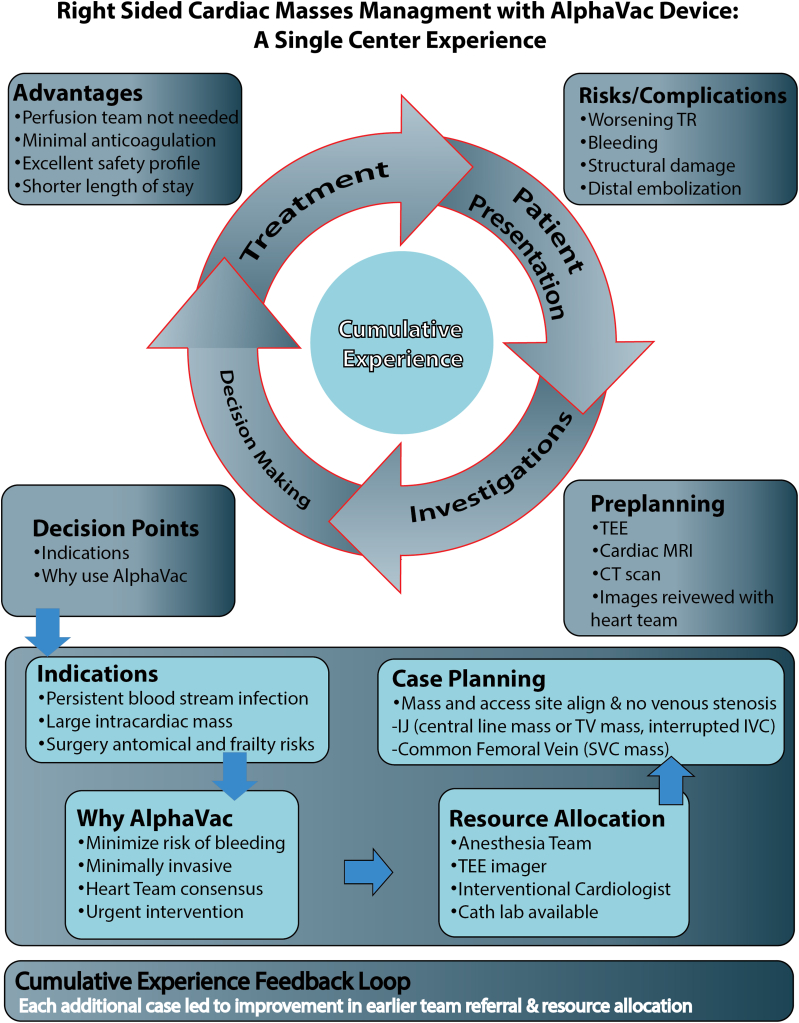
**Right sided cardiac masses management with AlphaVac device: a single center experience.** CT, computed tomography; IJ, internal jugular; IVC, inferior vena cava; MRI, magnetic resonance imaging; SVC, superior vena cava; TEE, transesophageal echocardiogram; TR, tricuspid regurgitation; TV, tricuspid valve.

Our case series involves consecutive patients who presented with right-sided cardiac chamber masses who were treated with AlphaVac. The overall clinical workflow and team-based decision pathway are outlined in Figure 1. The urgency of presentation and severity of illness warranted an endovascular approach. In our experience, AlphaVac can serve as (1) a temporizing measure before complementary treatment, as in our second and sixth patients; (2) a stabilizing therapy, as in the fifth case; or (iii) a definitive treatment, as demonstrated in cases 3 to 5.

Percutaneous mechanical aspiration is used for acute pulmonary embolism management. Its application has expanded to include right atrial thrombi and IE management, as noted in the 2023 European Society of Cardiology guidelines, for which it was awarded a class IIb recommendation for right-sided IE treatment.^3^ The American Heart Association also endorsed mechanical thrombectomy as a potential therapy for right-sided IE.^4^ Compared with surgical embolectomy, PMA offers a shorter procedure time and lower surgical risk.^5^

AlphaVac allows precise aspiration while minimizing blood loss and the risk of damaging cardiac structures.^6^ It eliminates the need for a perfusionist, simplifying setup and reducing clinical resource demands.^6^ However, potential complications include vascular injury from large-bore access, hemorrhage or transfusion need, thrombus embolization, arrhythmias, cardiac perforation, and embolic stroke in patients with a patent foramen ovale.^7^

In our series, AlphaVac was successfully utilized to remove intracardiac vegetations and thrombi in patients requiring urgent intervention or deemed unsuitable for surgery. It enabled bridging to definitive management. In case 3, approximately 60% of a right atrial wall–attached mass was removed, reducing thromboembolic risk when surgery was unfeasible due to anatomic constraints. Two patients had acute blood loss precluding anticoagulation, rendering AngioVac unsuitable. Procedural events in case 6 included concomitant aortic valve endocarditis with cusp perforation that eluded initial diagnosis owing to a lack of transesophageal echocardiogram secondary to esophageal stenosis, ultimately necessitating delayed double-valve surgical replacement. Overall, AlphaVac offered a practical option for stabilization in high-risk patients, although careful case selection and multidisciplinary evaluation remain essential.

## Conclusion

Our case series contributes to the limited literature on the AlphaVac system, demonstrating its efficacy and safety for various right heart masses. Being significantly less resource-intensive than the AngioVac system, and with a setup familiar to the current generation of large-bore endovascular device operators, it is uniquely suited to wide adoption in carefully selected patients. However, high-quality, larger registry data are needed to understand and develop guidelines and best practices.
