# Supplementary material for: Management of Right-Sided Cardiac Masses With Manual Aspiration Using AlphaVac Device: A Single-Center Case Series
Source: J Soc Cardiovasc Angiogr Interv. 2026 Jan 15;5(2):104160. doi: 10.1016/j.jscai.2025.104160 (PMC12923346; doi:10.1016/j.jscai.2025.104160)
Supplement: Video 1 [file mmc1.pptx]

## Slide 1
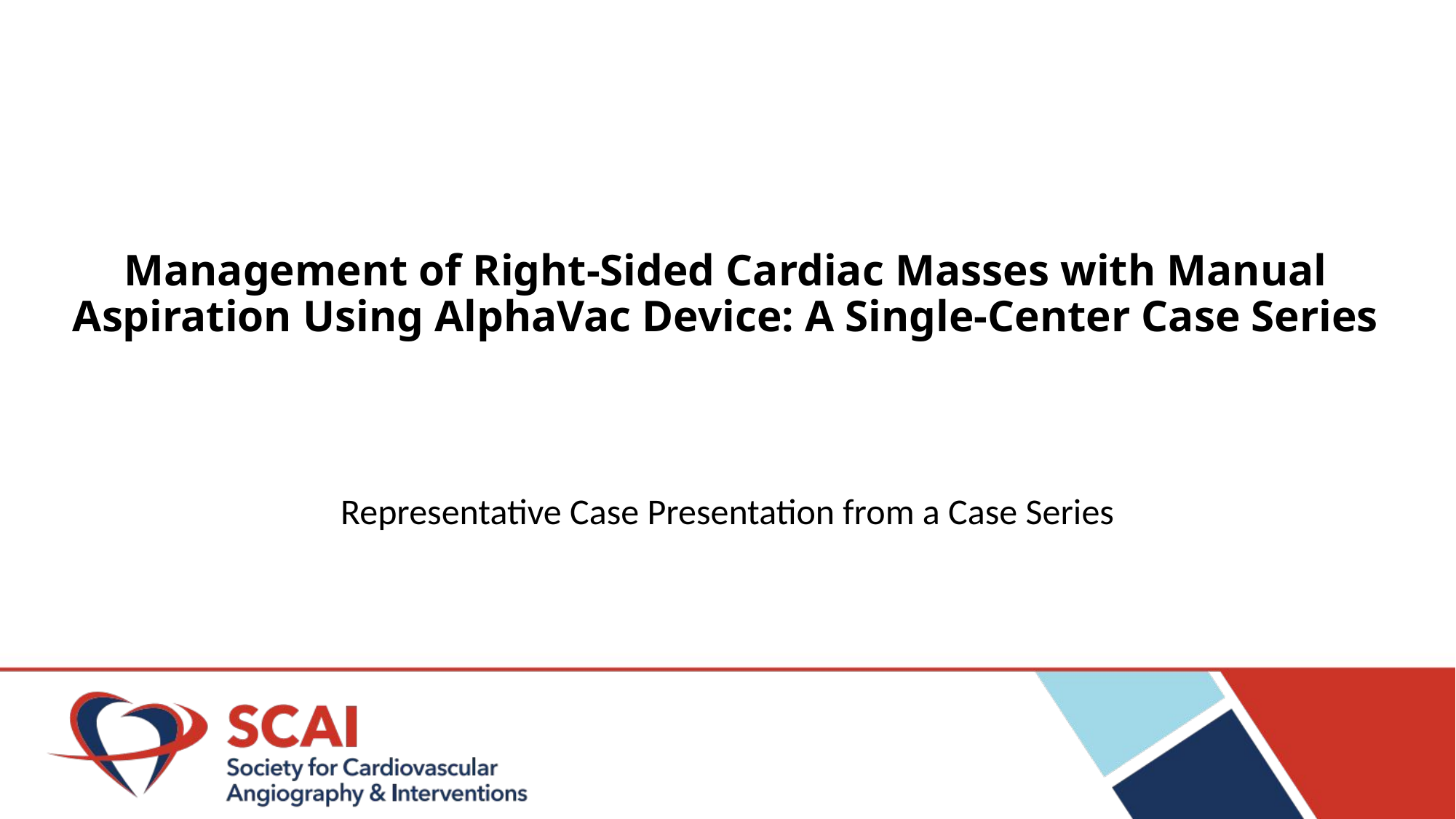

# Management of Right-Sided Cardiac Masses with Manual Aspiration Using AlphaVac Device: A Single-Center Case Series
Representative Case Presentation from a Case Series

## Slide 2
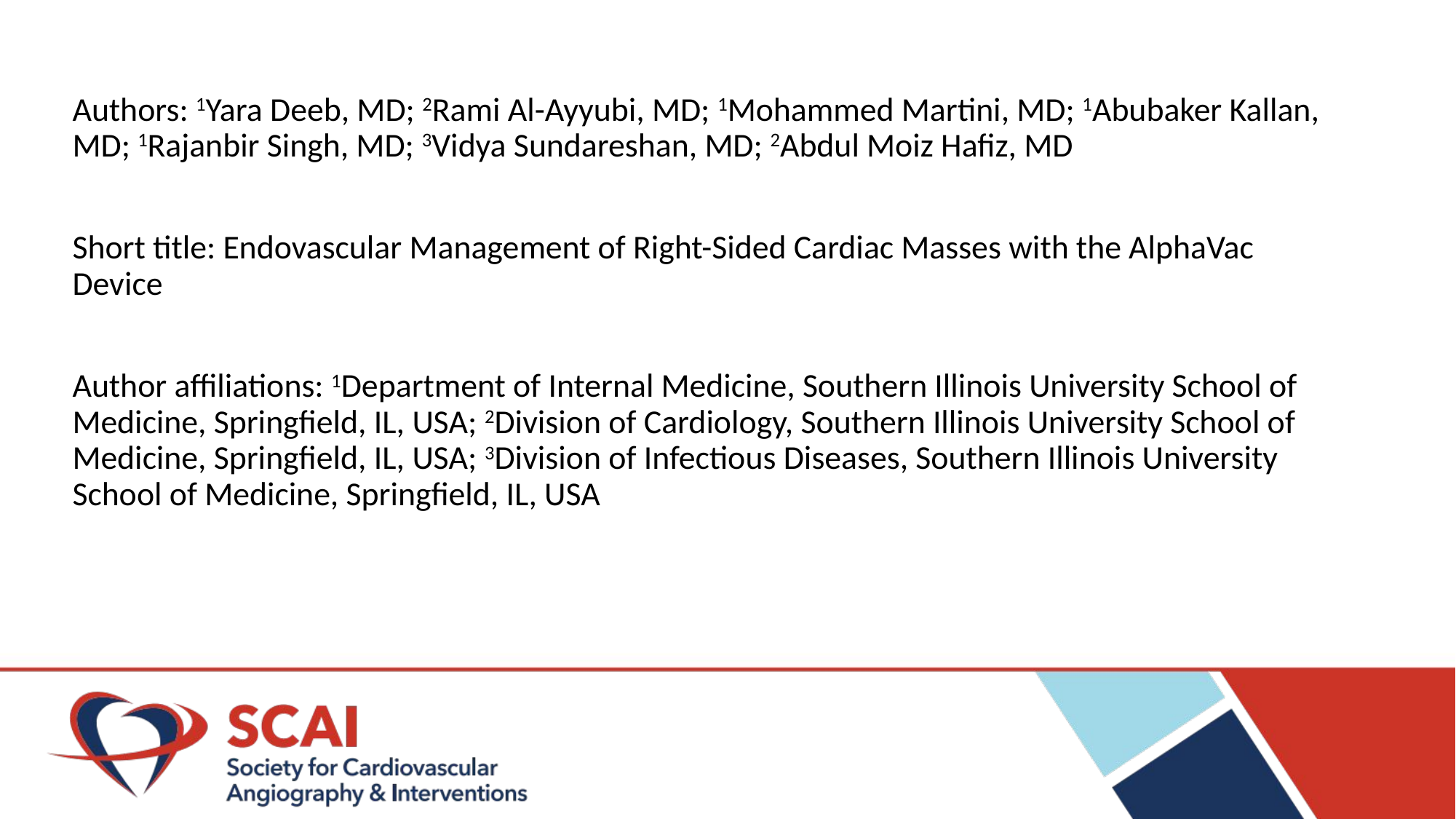

Authors: 1Yara Deeb, MD; 2Rami Al-Ayyubi, MD; 1Mohammed Martini, MD; 1Abubaker Kallan, MD; 1Rajanbir Singh, MD; 3Vidya Sundareshan, MD; 2Abdul Moiz Hafiz, MD
Short title: Endovascular Management of Right-Sided Cardiac Masses with the AlphaVac Device
Author affiliations: 1Department of Internal Medicine, Southern Illinois University School of Medicine, Springfield, IL, USA; 2Division of Cardiology, Southern Illinois University School of Medicine, Springfield, IL, USA; 3Division of Infectious Diseases, Southern Illinois University School of Medicine, Springfield, IL, USA

## Slide 3
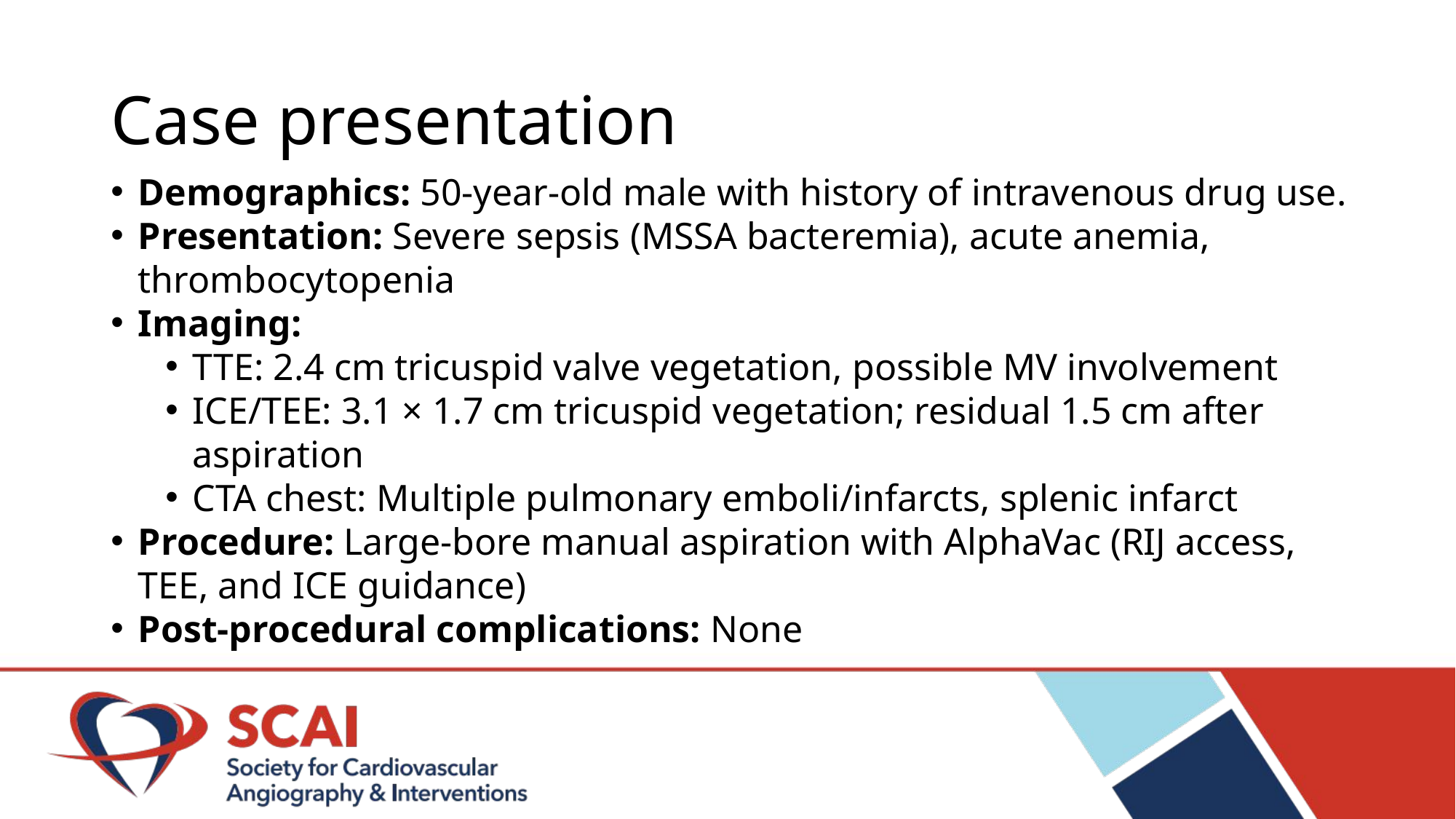

# Case presentation
Demographics: 50-year-old male with history of intravenous drug use.
Presentation: Severe sepsis (MSSA bacteremia), acute anemia, thrombocytopenia
Imaging:
TTE: 2.4 cm tricuspid valve vegetation, possible MV involvement
ICE/TEE: 3.1 × 1.7 cm tricuspid vegetation; residual 1.5 cm after aspiration
CTA chest: Multiple pulmonary emboli/infarcts, splenic infarct
Procedure: Large-bore manual aspiration with AlphaVac (RIJ access, TEE, and ICE guidance)
Post-procedural complications: None

## Slide 4
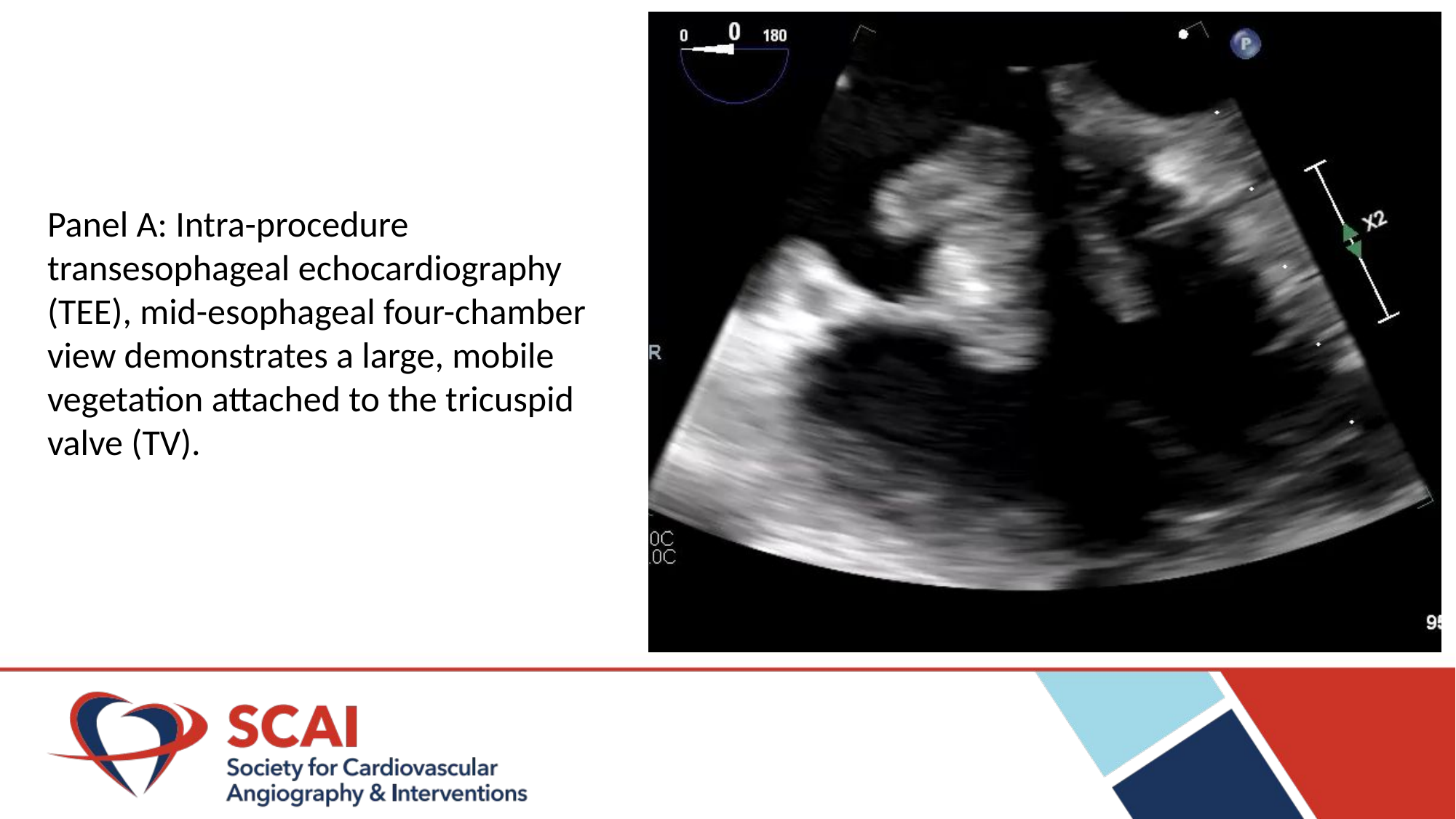

Panel A: Intra-procedure transesophageal echocardiography (TEE), mid-esophageal four-chamber view demonstrates a large, mobile vegetation attached to the tricuspid valve (TV).

## Slide 5
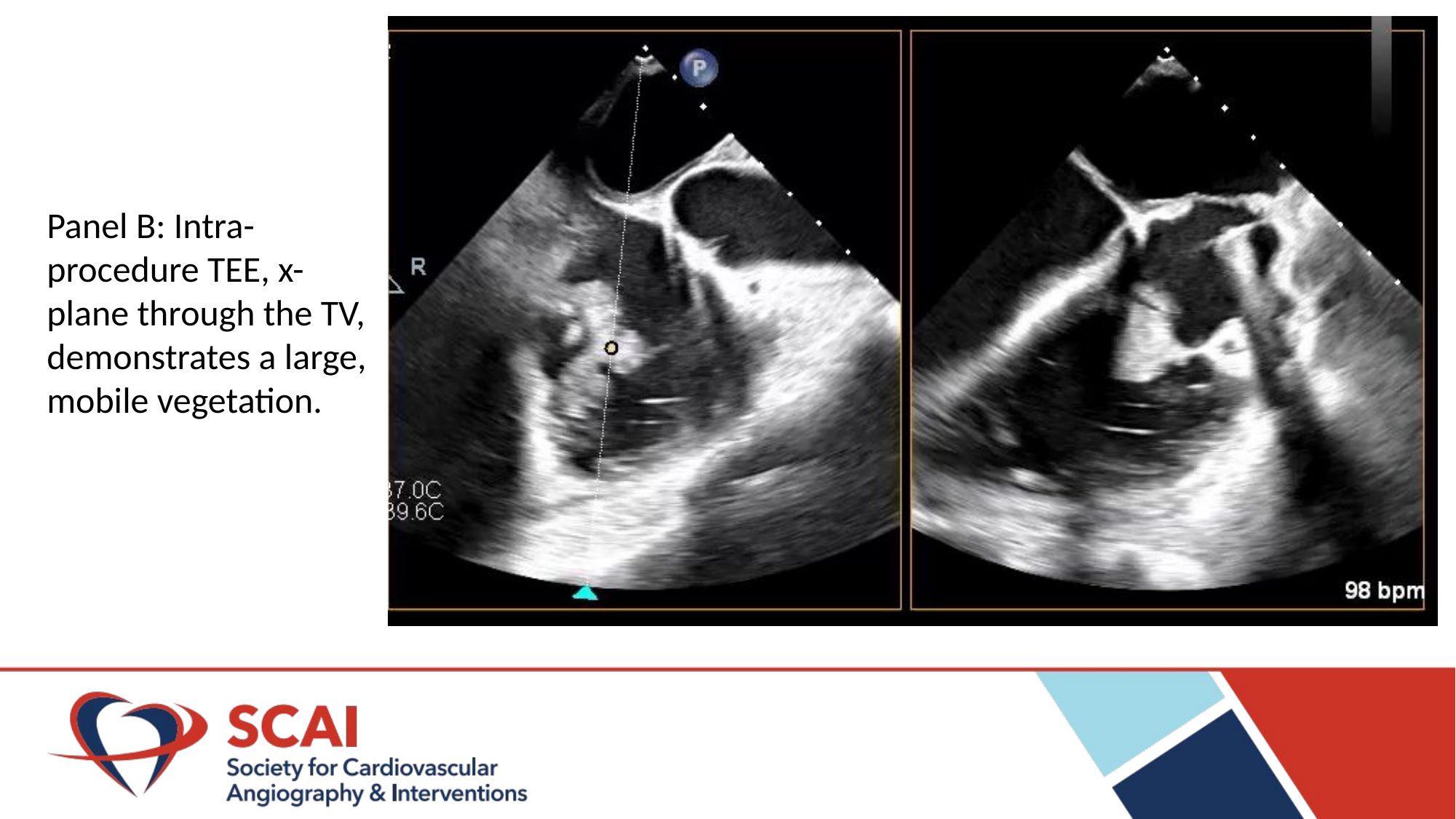

Panel B: Intra-procedure TEE, x-plane through the TV, demonstrates a large, mobile vegetation.

## Slide 6
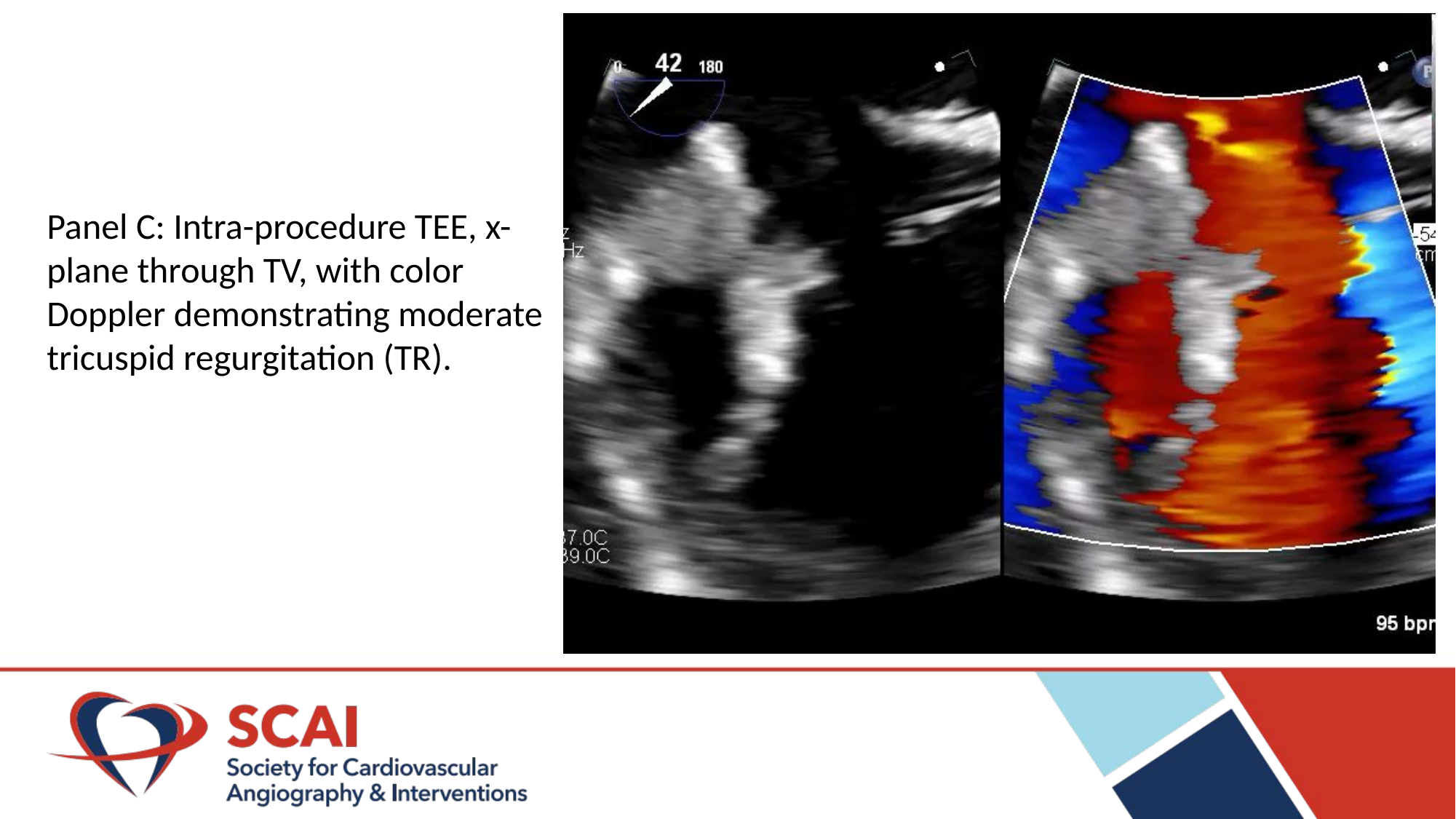

Panel C: Intra-procedure TEE, x-plane through TV, with color Doppler demonstrating moderate tricuspid regurgitation (TR).

## Slide 7
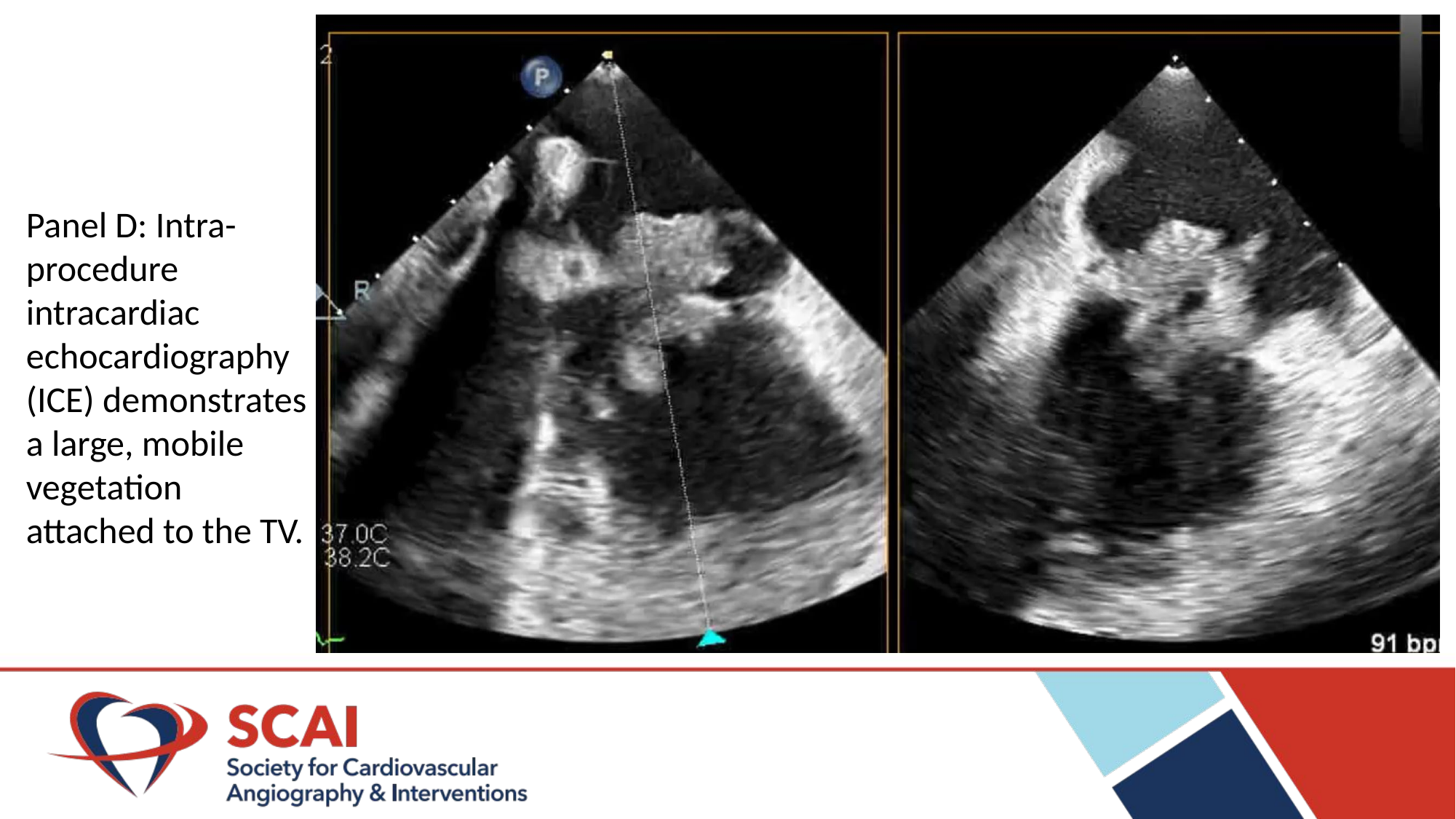

Panel D: Intra-procedure intracardiac echocardiography (ICE) demonstrates a large, mobile vegetation attached to the TV.

## Slide 8
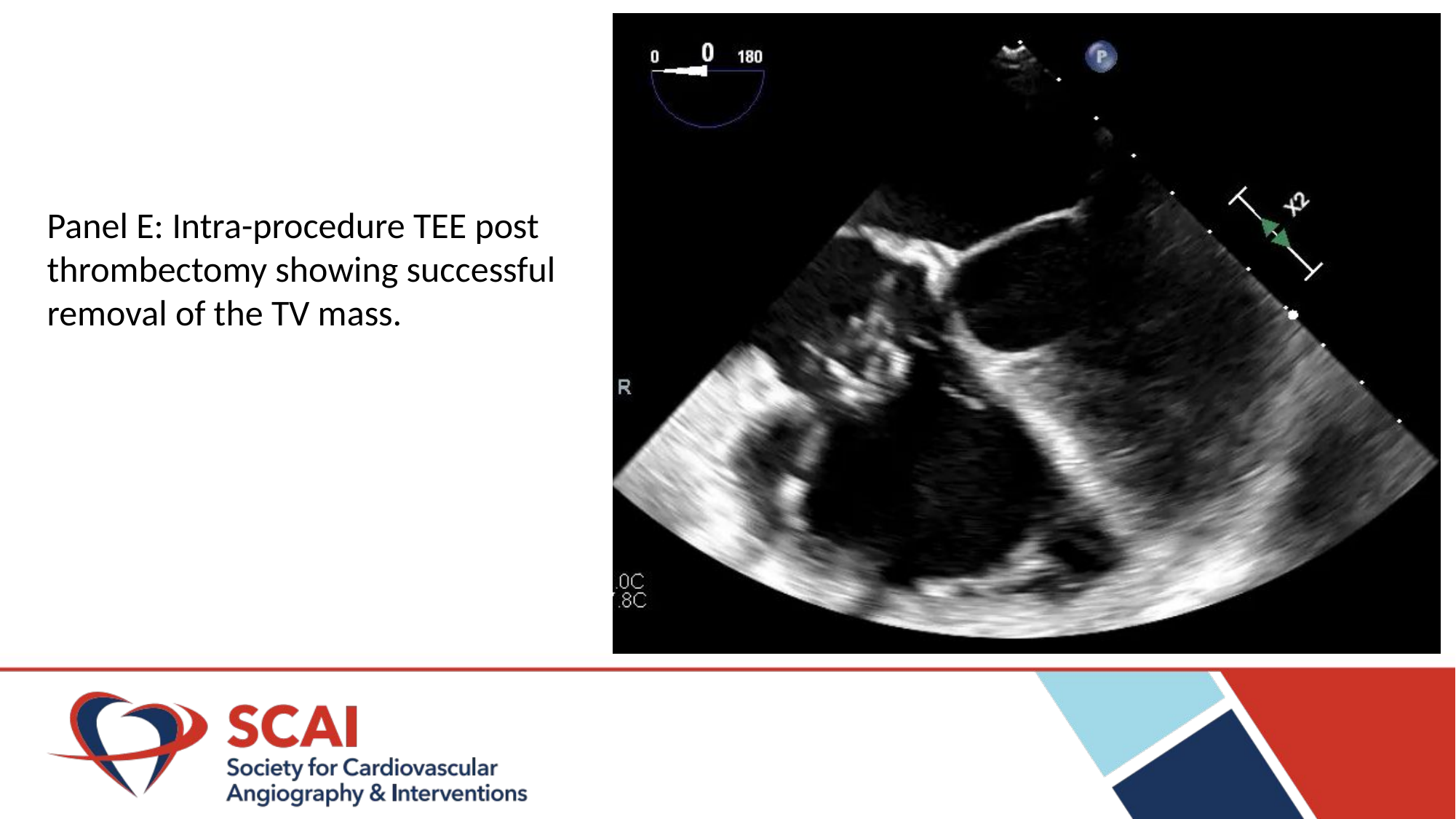

Panel E: Intra-procedure TEE post thrombectomy showing successful removal of the TV mass.

## Slide 9
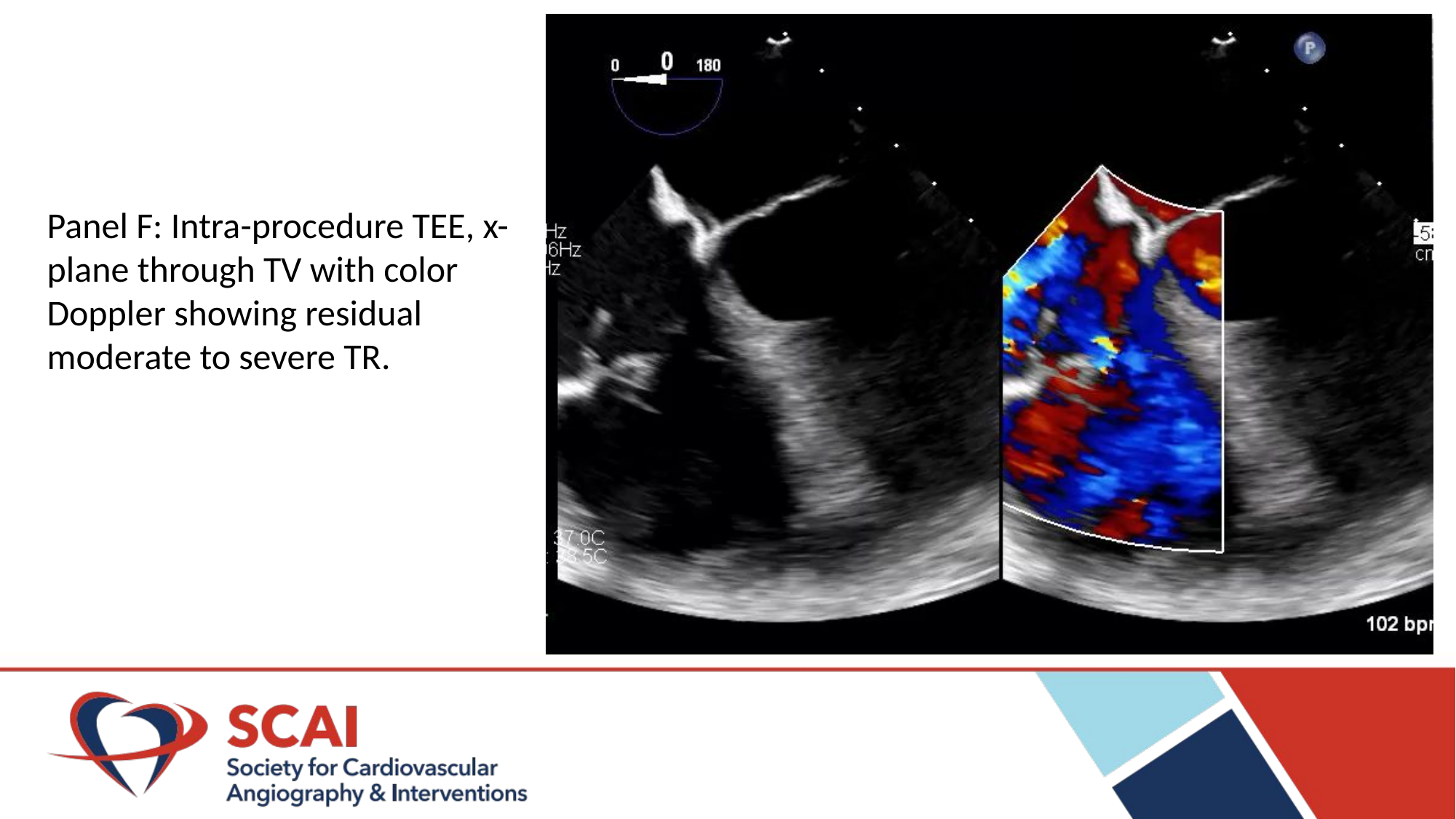

Panel F: Intra-procedure TEE, x-plane through TV with color Doppler showing residual moderate to severe TR.

## Slide 10
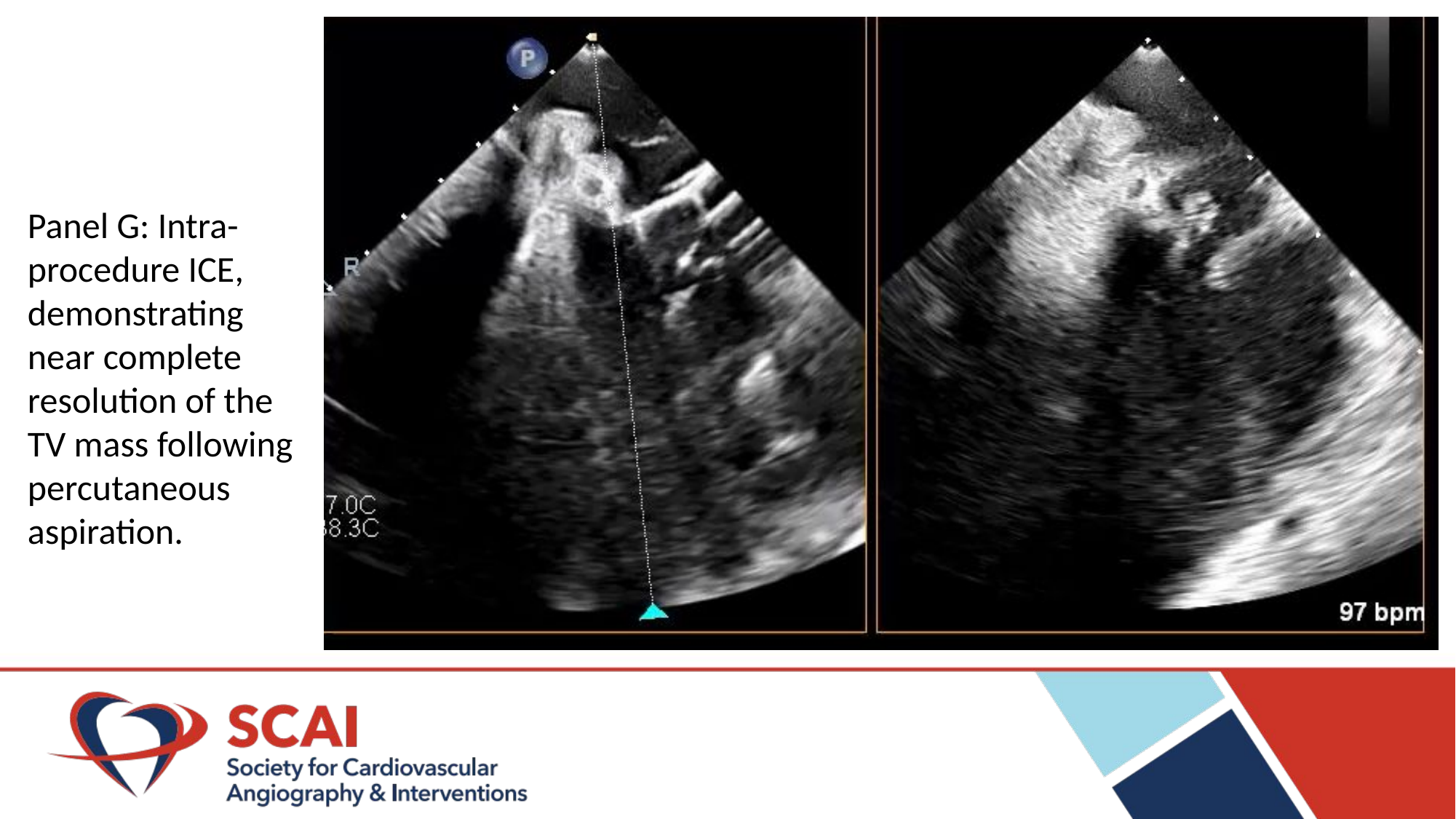

Panel G: Intra-procedure ICE, demonstrating near complete resolution of the TV mass following percutaneous aspiration.

## Slide 11
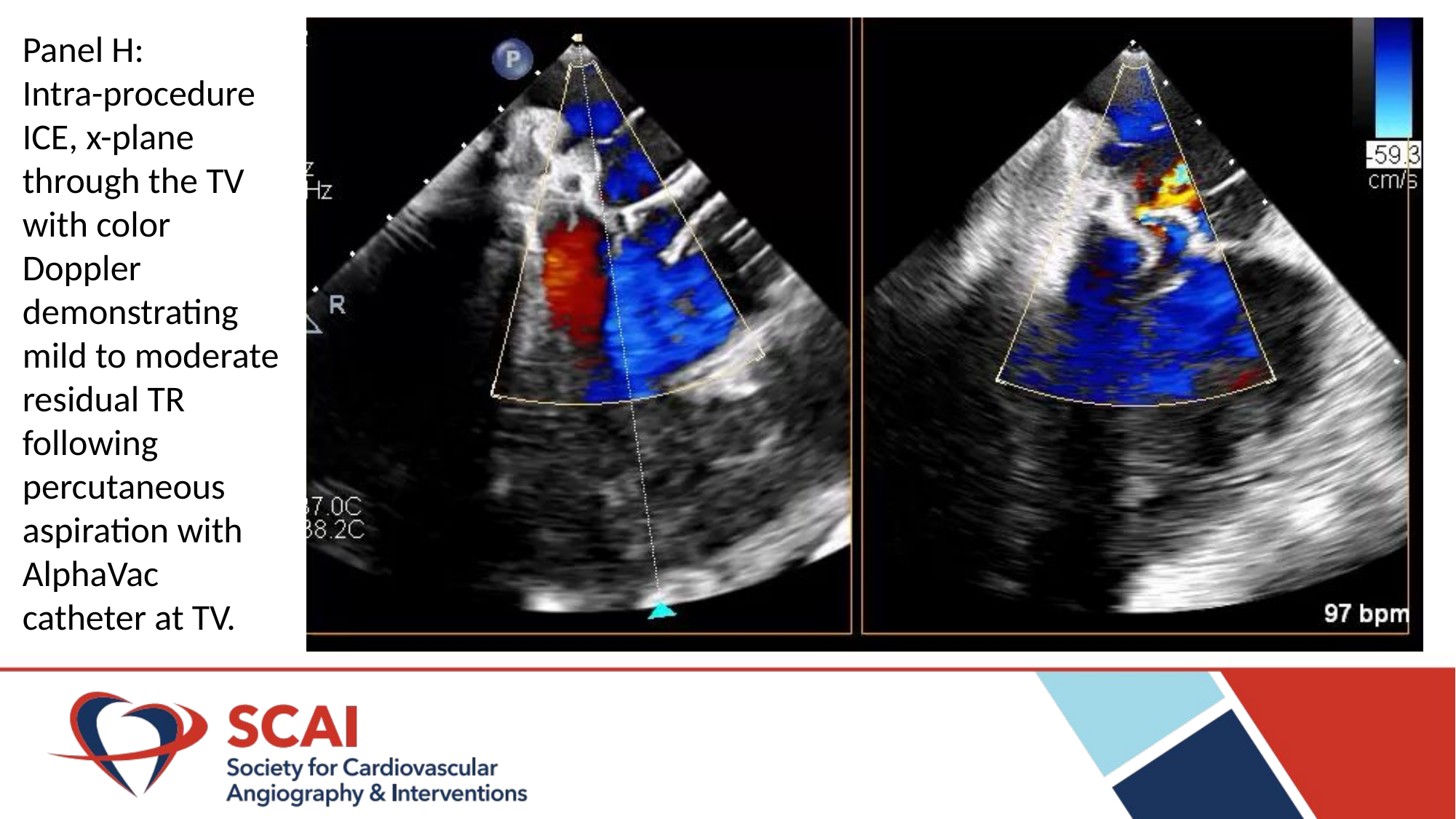

Panel H:
Intra-procedure
ICE, x-plane through the TV with color Doppler demonstrating mild to moderate residual TR following percutaneous aspiration with AlphaVac catheter at TV.

## Slide 12
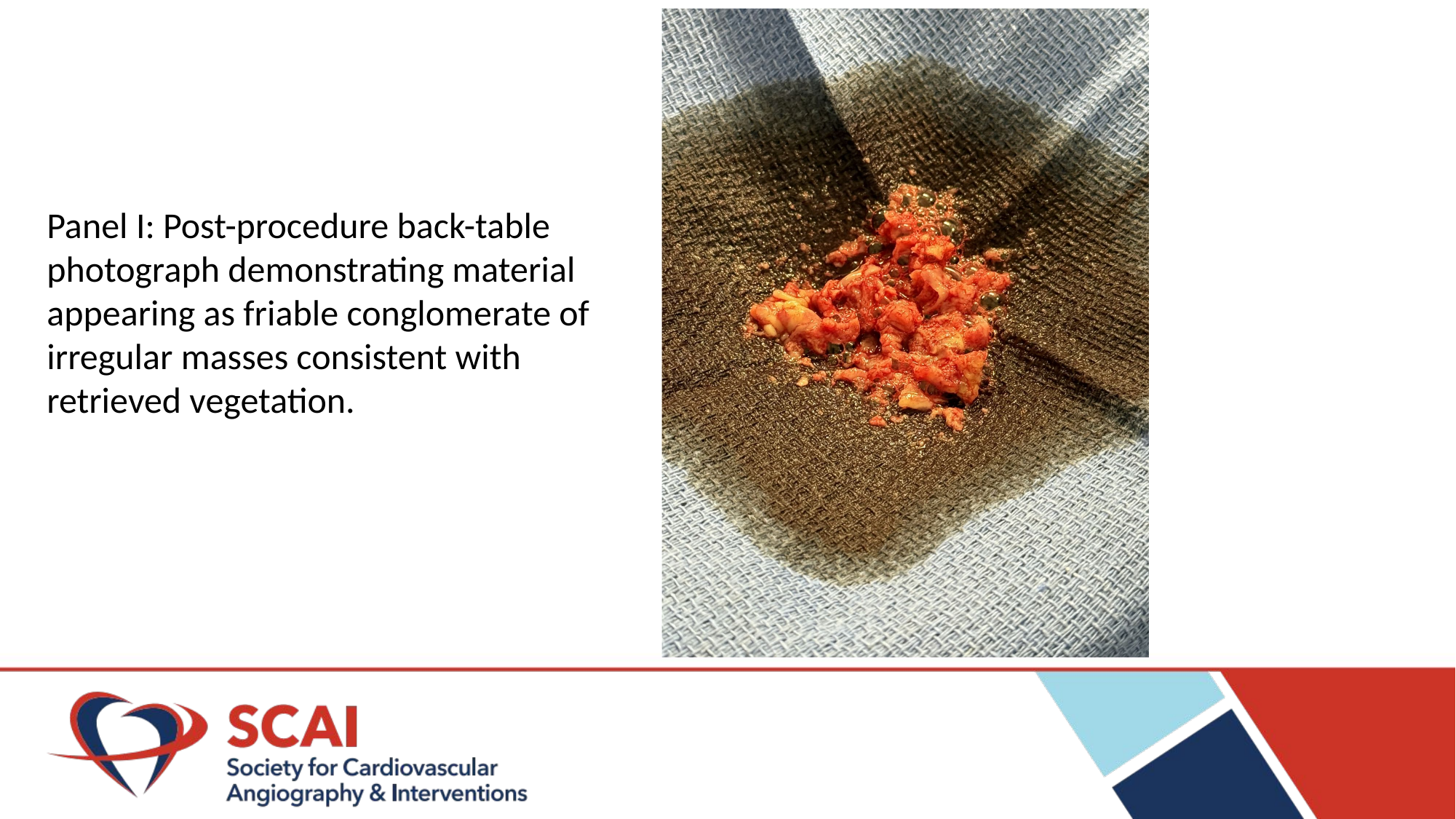

Panel I: Post-procedure back-table photograph demonstrating material appearing as friable conglomerate of irregular masses consistent with retrieved vegetation.

## Slide 13
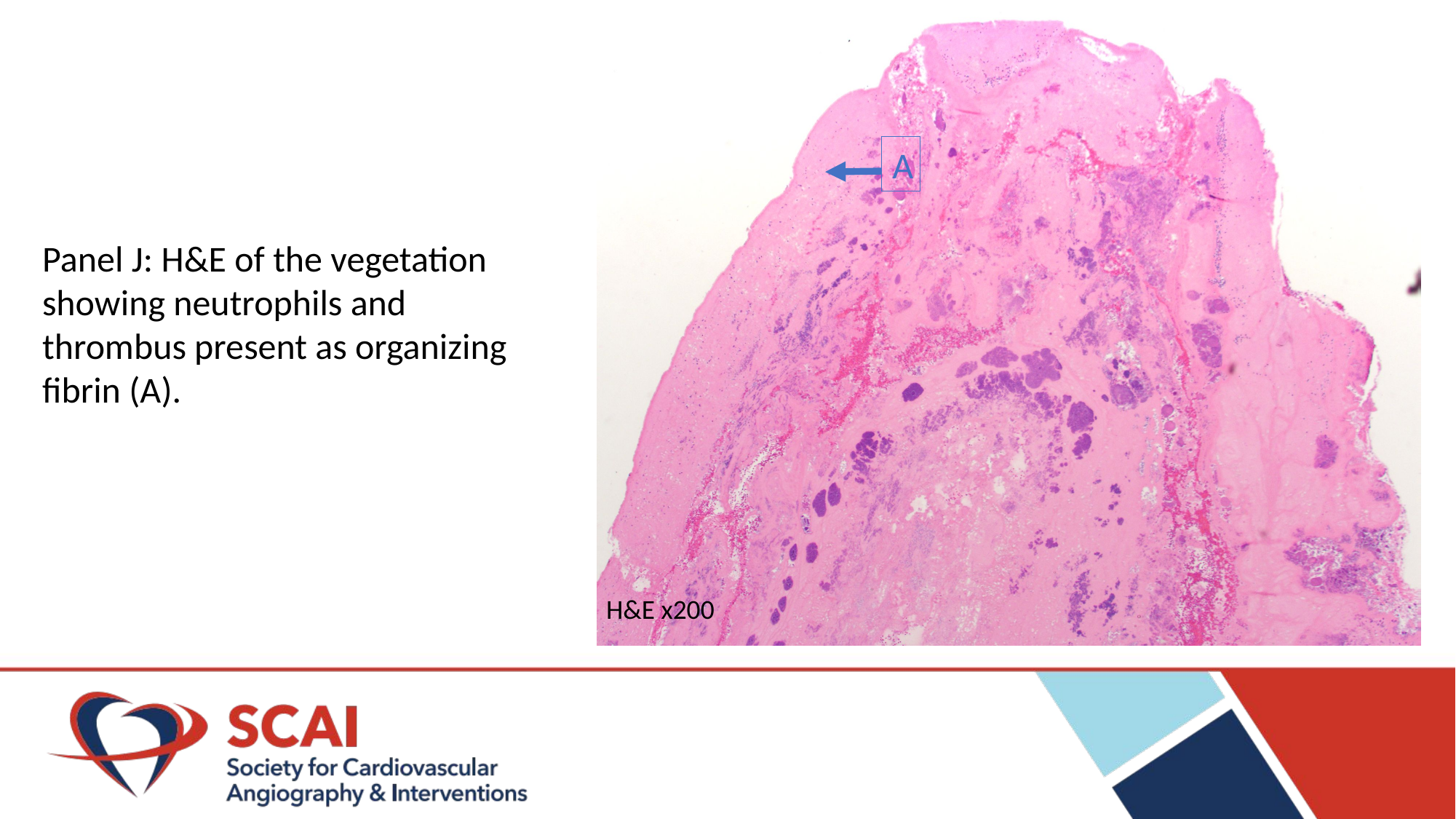

A
Panel J: H&E of the vegetation showing neutrophils and thrombus present as organizing fibrin (A).
H&E x200

## Slide 14
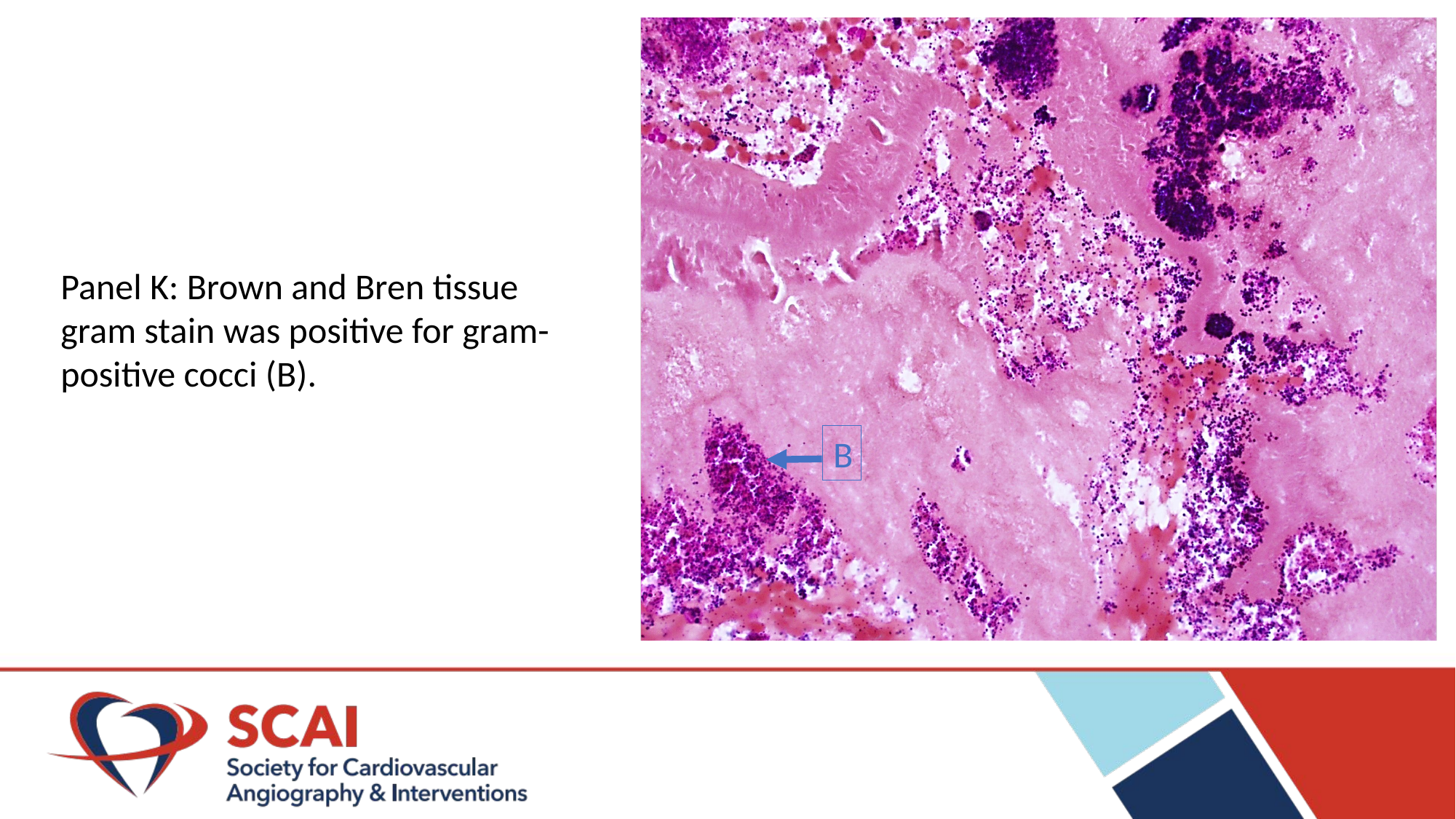

Panel K: Brown and Bren tissue gram stain was positive for gram-positive cocci (B).
B

## Slide 15
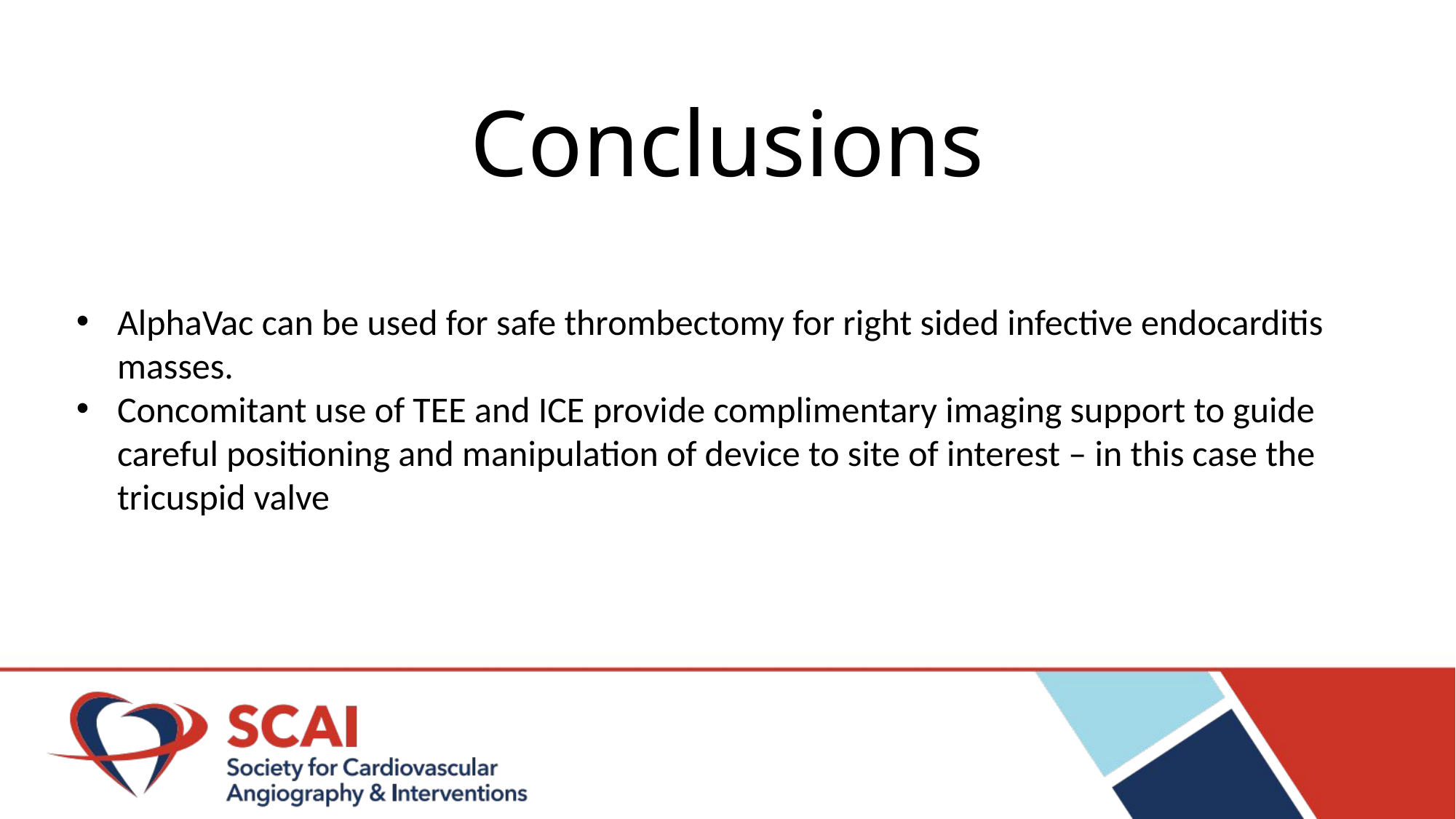

Conclusions
AlphaVac can be used for safe thrombectomy for right sided infective endocarditis masses.
Concomitant use of TEE and ICE provide complimentary imaging support to guide careful positioning and manipulation of device to site of interest – in this case the tricuspid valve
